# Apheresis in Neurological Disorders

**DOI:** 10.3390/jcm9103211

**Published:** 2020-10-06

**Authors:** Johannes Dorst

**Affiliations:** Department of Neurology, University of Ulm, Oberer Eselsberg 45, 89081 Ulm, Germany; johannes.dorst@uni-ulm.de; Tel.: +49-731-177-5285

## 1. Introduction

Plasma exchange (PE) and immunoadsorption (IA) constitute important options in the treatment of various autoimmune disorders across different medical disciplines. Their pathophysiological rationale is mainly based on the removal of auto-antibodies and a beneficial modulation of the immune system. From a theoretical point of view, apheresis offers an attractive therapeutical option since its effect relies on eliminating pathogenic components rather than administering drugs which may cause significant side effects. Neurological indications include, amongst others, steroid-refractory relapse of multiple sclerosis (MS), myasthenia gravis, autoimmune encephalitis (AE), Guillain–Barré syndrome (GBS), and chronic inflammatory demyelinating polyneuropathy (CIDP). Although frequently applied in clinical practice, evidence regarding efficacy and safety for the use of PE and IA in the aforementioned indications is generally low, which is directly related to the fact that drugs and medical devices are handled differently with regard to regulatory approvals in most countries, i.e., adequate, indication-specific phase III studies are generally not required in order to introduce medical devices into clinical practice. Therefore, little is known about the efficacy of PE and IA compared to each other and compared to other treatment options. Likewise, knowledge about optimal treatment regimens for conduction of PE and IA is completely lacking.

Therefore, this Special Issue of the Journal of Clinical Medicine focuses on articles which either present novel original data improving the evidence for efficacy and safety of PE and IA in specific neurological indications or review the existing literature in order to understand their significance in various autoimmune neurological diseases and to provide recommendations for their use in clinical practice. Furthermore, various articles highlight potential future areas of application, even beyond classical autoimmune-mediated diseases.

### 1.1. Methodological Differences between Plasma Exchange and Immunoadsorption

Although both PE and IA primarily focus on removing auto-antibodies from the blood, it is important to understand that both methods imply further immune-modulating mechanisms, including up- and down regulation of anti- and pro-inflammatory proteins [1] and potentially other alterations not yet explored. While, in PE, the plasma including all proteins is removed and substituted by human albumin or fresh frozen plasma, IA is more selective, mainly removing immunoglobulins while largely sparing other plasma constituents. Therefore, theoretically speaking, IA is supposed to offer a low-risk alternative compared to PE, since the preservation of coagulation factors should imply fewer bleeding complications, and since no volume replacement solution is needed, a lower risk of allergic reactions has to be expected. On the other hand, evidence for efficacy for IA is even lower compared to PE for many indications, which does not necessarily imply inferiority compared to PE, but may be simply explained by the fact that IA is the most recent method, and therefore, fewer clinical trials have been performed. Furthermore, preservation of certain pro-inflammatory proteins might compromise the efficacy of IA compared to PE, which may be a concern especially in autoimmune diseases like MS and CIDP, in which distinct disease-related auto-antibodies have not been characterized in the majority of patients. As long as the immunological mechanisms underlying both the diseases and the treatments are not fully understood, therapeutic decisions solely depend on the outcomes of clinical studies comparing different treatment options.

### 1.2. The Importance of Specific Treatment Regimens

While at least some studies to date have addressed the question of whether to prefer IA or PE in various indications, the question of which specific treatment regimen offers the best ratio between efficacy and safety has so far been completed neglected. Theoretically speaking, a wide array of treatment regimens is possible for both PE and IA with regard to plasma volumes (PVs) processed per treatment, number and frequency of treatments, time intervals between treatments, and peri-procedural medication such as antibiotics, anticoagulants, immunoglobulins, and volume substitution solutions. Due to missing evidence, generally accepted guidelines are lacking, and treatment regimens are therefore chosen based on local expertise and preference. The importance of considering specific treatment regimens is highlighted by our data presented in this Special Issue [2], which show that the advantages of IA compared to PE regarding safety and tolerability, as previously described [3], completely disappear when reducing the PV exchanged during each session in PE. On the other hand, it has, of course, to be questioned if a low-volume PE is equally effective. These questions can only be addressed by future comparative randomised controlled trials (RCTs).

### 1.3. Evidence for the Use of Plasma Exchange and Immunoadsorption in Specific Indications

In this Special Issue, several articles address the use of PE and/or IA in specific auto-immune mediated neurological diseases by either systematically analysing the existing literature or presenting original data.

#### 1.3.1. Multiple Sclerosis

PE constitutes an established treatment option in MS, mainly based on the RCT published by Weinshenker et al. in 1999 [4] which found a superiority of PE compared to sham treatment in patients with steroid-refractory relapse. Although this study is well done and represents one of the very few examples of an RCT investigating the efficacy of apheresis, the low number of subjects (*n* = 22) must be kept in mind when interpreting the results. In 2019, we finalized an RCT comparing PE and IA in 60 patients with steroid-refractory relapse [5]. In this study, patients in both groups showed significant improvements of clinical outcome parameters. Although PE patients responded faster, IA patients showed significantly larger improvements after 4 weeks, indicating a potential superiority of IA. In this study, we found no difference with regard to safety. In this Special Issue, Mark Lipphardt and colleagues present a systematic meta-analysis [6] including all observational studies and RCTs to date. They found response rates of 76.6% for PE and 80.6% of IA, indicating an about equal and good efficacy for both methods, while safety was also equal.

Steffen Pfeuffer and colleagues investigated a related, equally important question: should another ultra-high-dose methyl-prednisolone (MP) therapy be interpolated after an initial, unsuccessful high-dose MP treatment, or should apheresis be performed directly? In their retrospective database study in 145 patients [7], they found a surprisingly clear result in favour for the direct apheresis (PE) approach, which seriously questions the recommendations of many national and international guidelines and highlights the importance for a sufficiently powered RCT addressing this issue.

Finally, Leoni Rolfes and colleagues provided a comprehensive review about the topic [8] including special situations like treatment of children and pregnant women.

#### 1.3.2. Immune-Mediated Neuropathies

GBS and CIDP constitute the most important autoimmune-mediated neuropathies. GBS represents the acute form and is generally treated with either PE or intravenous immunoglobulins (IVIg) based on several RCTs and a recent Cochrane review [9] which, in summary, suggest equal efficacy. Evidence regarding the efficacy of IA is rather low and mainly relies on retrospective case reports and series. CIDP is often considered as the chronic form of GBS, although it features strong heterogeneity with regard to clinical symptoms and natural courses, which makes treatment and its evaluation difficult. MP, IVIg, and PE are generally considered as the main treatment options of CIDP, and there is no convincing evidence of which approach should be preferred with regard to efficacy and safety. Additionally, immunosuppressive drugs such as azathioprine and rituximab are sometimes used in therapy-refractory cases. Regarding IA, one small RCT suggested superior short-term effects compared to PE [10], while we have demonstrated recently that repeated IA may offer a promising long-term treatment option in therapy-refractory, chronic progressive cases [11].

Although to date distinct disease-related antibodies are not detected in the majority of patients, the discovery of potential pathogenic auto-antibodies against proteins of the node of Ranvier and paranodal regions [12] have corroborated the importance of auto-antibodies in at least a subgroup of patients with immune-mediated neuropathies and therefore supported the rationale to apply treatments which target these antibodies. Alexander Davies and colleagues [13] investigated whether the existence of such antibodies may predict the response to apheresis in patients with GBS and CIDP. Interestingly, they did not find a clear correlation between the presence of known auto-antibodies and treatment response, suggesting that further, undiscovered antibodies may be present.

#### 1.3.3. Autoimmune Encephalitis

The term autoimmune encephalitis (AE) refers to a group of diseases which are characterized by antibodies against neuronal surfaces and synaptic proteins and feature a large spectrum of clinical pictures, including focal neurological, psychiatric, and cognitive symptoms. As opposed to the aforementioned indications, specific auto-antibodies against proteins like N-methyl-D-aspartate (NMDA), gamma-aminobutyric acid (GABA), leucine-rich, glioma inactivated protein 1 (LGI1), and many others have been described and associated with typical clinical presentations. Since high-level evidence is lacking, no universally accepted treatment standards exist. Besides apheresis, current therapeutic strategies include high-dose MP, IVIg, and long-term immunosuppressive drugs such as cyclophosphamide and rituximab. IA is increasingly recognized as a promising therapeutic approach in AE and is even considered as a first-line option in many centers. In their article [14], Rosa Rössling and Harald Prüss review the most relevant studies regarding the use of therapeutic apheresis in AE. Based on their evaluation, they conclude that apheresis constitutes a promising treatment option in AE which should be applied early after disease onset, while they found no evidence that prior treatment with IVIg or steroids yields any additional advantages. Furthermore, they found a clear benefit for patients with antibodies against surface or synaptic antigens, while the effect on patients with onco-neuronal antibodies is less clear.

#### 1.3.4. Chronic Fatigue Syndrome

Myalgic encephalomyelitis / chronic fatigue syndrome (ME/CFS) is a disease characterized by severe fatigue as well as various cognitive, autonomic, and immunological symptoms. The pathophysiological background seems to be complex and has not been fully understood to date. Various auto-antibodies have been described to be associated with the disease, most notably antibodies against the muscarinic acetylcholine receptors (MAR) and β_2_-adrenoreceptors (β_2_AR). In their previous pilot study [15], Carmen Scheibenbogen and colleagues reported that 7/10 patients with ME/CSF showed rapid clinical improvement and significant reductions of β_2_AR antibodies after IA therapy. In this special issue, they present follow-up data from 5 patients who had previously responded to IA treatment and who underwent a subsequent, adjusted IA protocol 2 years later [16]. They found a positive response in 4/5 patients, confirming their previous results that IA may constitute a viable treatment option in ME/CSF, although further controlled studies with higher numbers of patients are certainly needed.

#### 1.3.5. Potential Future Indications and Outlook

This Special Issue also features two articles which focus on potential future areas of application, including diseases which are not generally considered to feature pronounced autoimmune-mediated mechanisms.

Stefan Kayser and colleagues review potential indications for C-reactive protein (CRP) apheresis [17], aiming at removing CRP from the patients’ blood by using specific adsorbers. The pathophysiological background of this approach is based on the finding that CRP—most commonly known as an inflammatory biomarker—in fact plays an important role in immunological processes itself by mediating phagocytosis of damaged cells. Under certain conditions including myocardial infarction and ischemic stroke, these mechanisms may have negative impacts as they imply destruction of potentially salvageable tissue. The authors describe preliminary results from a multi-center trial on CRP apheresis in myocardial infarction as well as an upcoming trial in ischemic stroke. They also briefly touch on further potential indications for this interesting approach.

Finally, Sylvia Stracke and colleagues describe the IMAD trial (Efficacy of immunoadsorption for treatment of persons with Alzheimer dementia and agonistic autoantibodies against alpha 1A-adrenoceptor) [18] which tests a novel therapeutic approach for Alzheimer dementia using immunoadsorption. The study is based on the finding that agonistic autoantibodies against α_1_- and β_2_-adrenoceptors are present in 50% of patients with dementia and expands on a previous small trial in 8 patients [19] which demonstrated that IA was safe and able to significantly reduce α_1_-adrenoceptor antibodies. Furthermore, the Mini Mental State Examination (MMSE) scores of these patients remained rather stable over the following 12–18 months. The IMAD trial aims at investigating the effects of IA on brain perfusion and disease progression on 15 patients with Alzheimer dementia and agonistic auto-antibodies.

## 2. Conclusions

The articles presented in this Special Issue reveal a broad spectrum of present as well as potential future indications for therapeutic apheresis and unanimously support the view that both PE and IA constitute promising, low-risk therapeutic options in the treatment of various autoimmune neurological diseases. However, they also highlight the need to improve indication-specific evidence, which can mainly be achieved by conducting sufficiently-powered RCTs which aim at comparing different treatment options. Along with a better understanding of underlying immunological processes and development of novel prognostic biomarkers, such studies will facilitate adequate therapeutic decision-making and eventually improve clinical outcomes of patients with autoimmune-mediated diseases.
